# Digital live imaging of intraoperative electrocochleography during cochlear implantation: the first 50 patients

**DOI:** 10.1007/s00405-023-08197-1

**Published:** 2023-08-30

**Authors:** Benedikt Höing, Theda Eichler, Viktoria Juelly, Moritz Meyer, Lea Jung, Laura Waschkies, Stephan Lang, Diana Arweiler-Harbeck

**Affiliations:** 1 Department of Otorhinolaryngology, Head and Neck Surgery, University Hospital Essen, University Duisburg-Essen, Hufelandstrasse 55, 45147 Essen, Germany; 2 Cochlear Implant Rehabilitation Centre Ruhr (CIC), Essen, Germany

**Keywords:** Intraoperative electrocochleography, Digital microscopic imaging, Cochlear implantation, Hearing preservation

## Abstract

**Introduction:**

Real-time visualization of intraoperative electrocochleography (ECochG) potentials via a digital microscope during cochlear implantation can provide direct feedback during electrode insertion. The aim of this prospective, randomized study of 50 patients was to obtain long-term data with a focus on residual hearing preservation and speech understanding.

**Material and methods:**

Cochlear implantations were performed in 50 patients (26 female, 24 male) with residual hearing using a digital microscope. Patients were randomized into two groups. Intraoperative ECochG potentials were either displayed directly in the surgeon’s field of view (picture-in-picture display, PiP) or not directly in the field of view (without picture-in-picture display, without PiP). Residual hearing preservation and speech comprehension were recorded within a 1-year follow-up period, compared between groups (PiP versus without PiP) and to a control group of 26 patients implanted without ECochG.

**Results:**

Mean insertion time was significantly longer in the picture-in-picture group (*p = *0.025). Residual hearing preservation after 6 weeks at 250 Hz was significantly better in the picture-in-picture group (*p = *0.017). After one year, 76% of patients showed residual hearing in the picture-in-picture group (62% without picture-in-picture technique, *p = *n.s.). Use of the picture-in-picture technique resulted in better long-term pure tone residual hearing preservation at 250, 500, and 1000 Hz. Speech intelligibility improved by 46% in the picture-in-picture group (38% without picture-in-picture).

**Discussion:**

This study is the first to describe long-term results in a large cohort of cochlear implant patients in whom digital visualization of intraoperative ECochG was used. Our results show that visualization of intraoperative ECochG has a positive effect on residual hearing preservation.

## Introduction

Cochlear implants (CI) are successfully implanted in patients with deafness or severe hearing loss and enable patients to receive sound. The surgically inserted electrode directly stimulates the human spiral ganglion cells [1]. Patients benefiting from cochlear implants are deafened infants [2, 3], elderly people [4], patients with Single-Sided Deafness [5] and, in the last decade, also patients with residual acoustic hearing. In the last years, hearing preservation during cochlear implantation has received more and more scientific and clinical attention. It could be shown that the combination of electric and acoustic stimulation of the cochlea by preserving residual hearing can have positive effects on speech understanding [6]. Additionally, perception of periodicity, sound localization [7, 8] and music perception [9] are improved. Potential mechanisms of hearing loss during cochlear implant surgery are suggested to be physical trauma in scala tympani and to the osseous spiral lamina, potential dislocations of the electrode to scala vestibuli and postoperative inflammation [10]. Therefore, methods to avoid intraoperative trauma are of high clinical interest. Advances in electrode design such as changes in stiffness, electrode tip, size and diameter, surface morphology or deployment mechanisms have contributed to a reduction of intraoperative damage to inner ear structures. Moreover, multiple intraoperative tests have been implemented. Impedance measurements, electrically evoked stapedius reflex threshold (eSRT) and electrically evoked compound action potentials (ECAP) or spread of neural excitation (SOE) provide useful and important information on correct position of electrode array and hearing nerve answers. These tests are broadly performed [11, 12]. However, a positive effect on hearing preservation or speech understanding has not been demonstrated [13] as they all measure the steady state after insertion of electrode and therefore don’t have a direct impact on the insertion itself.

A method enabling intraoperative monitoring of hearing preservation is electrocochleography (ECochG) [14]. In ECochG, electric potentials from inner and outer hair cells and auditory nerve caused by acoustic stimulation in the outer ear canal are detected [15, 16]. Measurement of those potentials can be performed in various ways, e.g. with an externally located electrode in the outer ear canal or a needle placed on the promontory [17, 18]. It is therefore a diagnostic instrument in preoperative screening before cochlear implantation. Used intraoperatively, it can give the surgeon direct feedback from the electrodes on the implant array monitoring the intracochlear activity. In case of deterioration, changes in insertion speed, insertion angle or applied pressure could be taken [19–21]. Moreover, retraction or stop of insertion is possible.

Digital microscopes such as the ARRISCOPE^®^ (Munich Surgical Imaging, Munich, Germany) can provide direct visualization of intraoperative measurements such as EcochG potentials during electrode insertion by picture-in-picture technique (PiP). The microscope captures the surgical field with a high-resolution imaging sensor, processes the image in real-time and displays the image in the binocular based on two small OLED displays. This digital processing chain enables the integration of additional data into the digital binocular. Feasibility of intraoperative ECochG during cochlear implantation using the digital microscope has been demonstrated [22].

The aim of this prospective randomized study is to demonstrate the postoperative audiometric results in the first 50 patients operated with the digital microscope using intraoperative ECochG. We were specifically aiming at comparing audiometric results and insertion time between patients who were operated on with picture-in-picture technique on the one side, to patients who were implanted without the picture-in-picture technique on the other side. In the latter, the surgeon got the information about the intraoperative ECochG-potentials via verbal feedback from the audiologist.

## Material and methods

### Preliminary setup in the lab

Prior to the intraoperative trial, a collaborative laboratory test was conducted to define and test the image exchange interface. In addition, the size of the picture-in-picture insert was adopted. As graphical input, the Cochlear™ Research Platform 1.1 running on a standard laptop (ThinkPad, Lenovo_TM_) was used. The laptop was connected to a Cochlear™ Nucleus^®^ 6 sound processor. A Slim Modiolar Practice Electrode was inserted by an experienced surgical support specialist (Cochlear) into a pre-drilled PHACON Temporal Bone Model. The Hybrid™ component acoustically stimulates the inner ear and the electrode array records the inner ear potential. The image signal was taken from the HDMI output of the laptop and fed via a converter (Blackmagic—Mini Converter UpDownCross HD) as HD-SDI signal (1080p 30/60 Hz) into a fully digital surgical microscope. The image signal of the Cochlear™ Research Platform 1.1 was displayed on all displays, including the binocular of the surgical microscope in full-screen mode or in thumbnail view (Picture-in-Picture mode). The user was able to switch between these two display modes using the handgrip keys. Depending on the selected display mode, the live image of the microscope was simultaneously shown on the same displays, either in full-screen or picture-in-picture mode. According to the testing surgeons it was easily possible to briefly switch attention between the insertion and the display of the Cochlear Research Platform.

### Patients

Fifty patients with residual hearing were included (26 female, 24 male). Mean age was 48.2 years (SD 21.4). Randomization in two groups was performed by a random generator (PiP: mean age 48.1 years, 15 female, 15 male; without PiP: mean age 44,5 years, 11 female, 9 male). Residual hearing preoperatively was defined as a hearing threshold of at least 80 dB HL in at least two tested frequencies (air conduction). Residual hearing postoperatively was defined as a hearing threshold of at least 115 dB HL in at least two tested frequencies (air conduction). Audiometric thresholds were measured in regular pre-diagnostic procedures before cochlear implantation. The mean pre-operative PTA_low_ was 68.3 dB HL (SD 23.7). Additionally, 26 patients (mean age: 48.1 years, 14 female, 12 male) were included for retrospective analysis of the data. All these patients were implanted with a CI622 electrode between 2019 and 2020 but without intraoperative ECochG measurements. The mean pre-operative PTA_low_ was 68.4 dB HL (SD 20.2).

### Cochlear implants

Commercially available Cochlear™ Nucleus^®^ Profile™ Plus with Slim Straight Electrode (CI622) (Cochlear^®^, Sydney, Australia) were used. Intraoperative ECochG was performed in all patients during electrode insertion. The patients were randomized into two groups: in group I potentials of intraoperative ECochG were visualized in the surgeon’s field of view. In group II potentials of intraoperative ECochG were not visualized in the surgeon’s field of view. Here, the audiologist gave acoustic feedback to the surgeon during insertion. In case of drops in the ECochG curve, the surgeon stopped the insertion and waited for the signal to recover.

### ***Intraoperative setting (***Fig. 1***)***

**Fig. 1:**
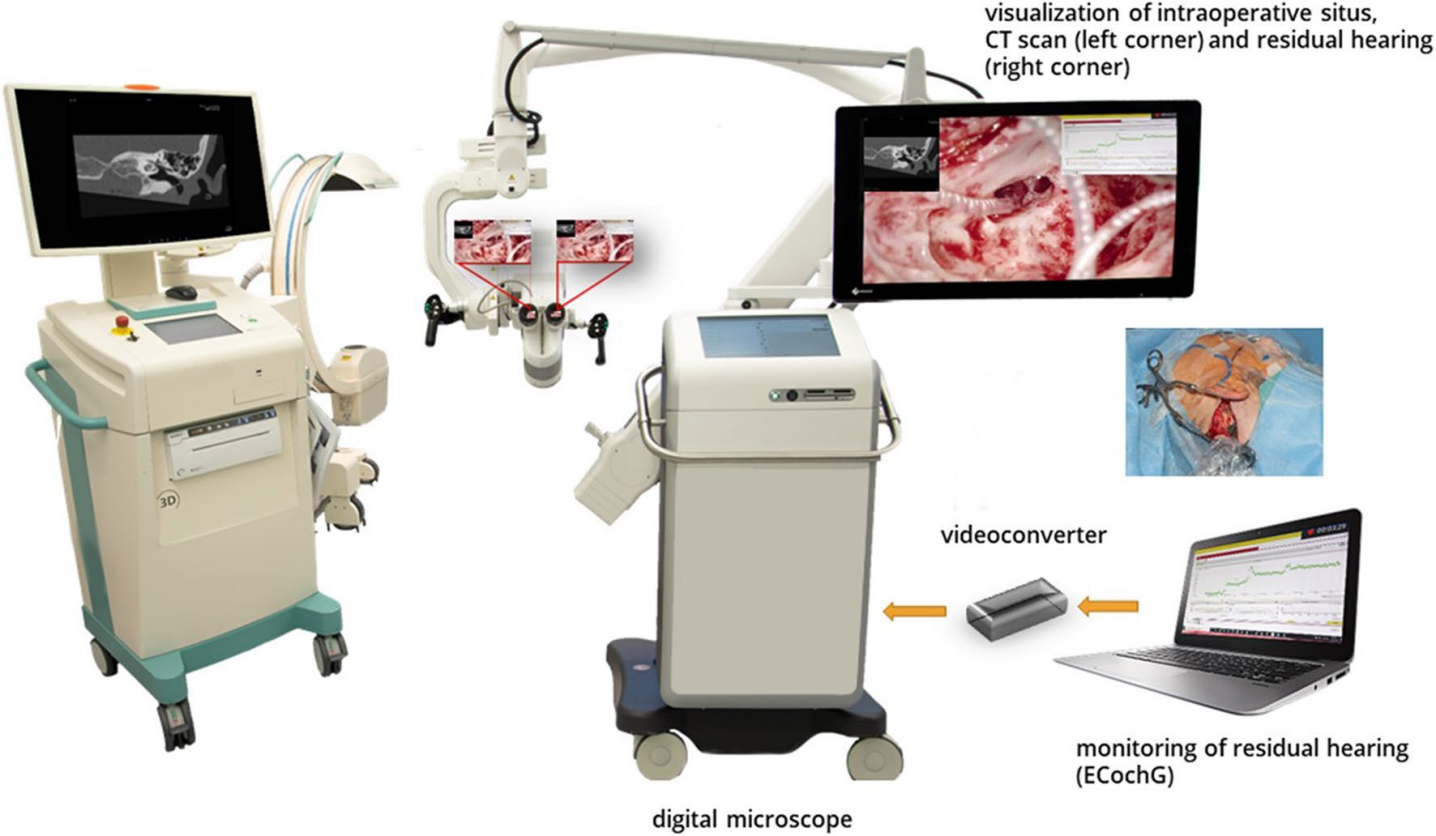
Intraoperative setting. Center of the intraoperative setting is the digital microscope. During electrode insertion, residual hearing is monitored by ECochG potentials measured as Cochlear microphonics which are transferred via a video converter from a laptop to the digital microscope and visualized in the top right corner in the surgeon’s field of view. Patients´ CT scans are displayed in the top left corner. Postoperative control for correct intracochlear positioning of the electrode is performed with C-arm X-ray-unit (left side)

The acoustic component of the sound processor was connected to a disposal sound tube (for 3 M E-A-RTONE™ insert earphones, 3 M AEARO) and a disposal ear-tip (3 M E-A-RLINK™, 3 M AEARO). The ear tip was positioned in the ear canal before surgery started. ECochG potentials were acoustically generated with a tone burst of 250 Hz, 112 dB HL and a duration of 7 ms. The screen of the Cochlear™ Research Platform 1.1 was transferred to the digital microscope ARRISCOPE^®^ (Munich Surgical Imaging, Munich, Germany) and appeared in the surgeon´s field of view. During the insertion of the electrode array, the amplitude of cochlear microphonics was continuously measured with 3 updates per second. For recording, the most apical electrode (E22) and the case electrode were used. Additionally, the amplitude growth function (AGF) resembling the intraoperative residual hearing was determined at the end of insertion at 250, 500, 750 and 1000 Hz.

### Audiometric tests

Preoperatively, all patients underwent standard tone and speech audiometry as well as objective hearing tests like auditory brainstem response (ABR) and cochlear microphonics (CM) in routine cochlear implant screening by professional audiometrists using Auritec audiometer (Auritec GmbH, Hamburg, Germany) and BERA Eclipse EP 25/ECochG/ASSR (Interacoustics A/S, Assens, Denmark). Additionally, air and bone conduction were measured one day before and one day after surgery. Postoperative monitoring of residual hearing and speech perception was performed at 6 weeks, 3 months, 6 months and 12 months postoperatively. All tests were performed according to the declaration of Helsinki and were approved by the local ethics committee. Informed written consent was obtained from all patients.

### Additional intraoperative monitoring during cochlear implant surgery

The electrode impedance mirrors the electrical resistance and gives additional information about a successful electrode array insertion and the technical integrity of the device. Electrical Stapedius Reflex Thresholds (eSRTs) are measured by visually observing the contraction of the stapedius muscle in the implant ear. Moreover, the function of CI and nerve was monitored by Electric Compound Action Potentials (ECAPs). Spread of Excitation (SOE) [11] and Trans-Impedance Matrix (TIM) measurement [23] identify and characterize the normal electrode position. Intraoperatively, the correct electrode position was examined with 3D C-arm X-ray-unit.

### Data analysis

Graphs and statistical analysis were performed using GraphPad Prism 6 (GraphPad Software Inc., LA Jolla, USA), SPSS (Ver. 27, IBM, Ehningen, Deutschland), MATLAB (Ver. R2022a, MathWorks Inc., USA) and Microsoft Excel (Redmond, USA). In the case of normal distribution, t-tests were obtained. If normal distribution could not be assumed, a Mann–Whitney-Wilcoxon test was applied.

## Results

### Insertion time

Insertion time was defined as the time between the first intracochlear electrode contact and the end of insertion. Mean insertion time in the picture-in-picture group was significantly longer than in the without picture-in-picture group (101 s versus 77.6 s, *p = *0.025, z = 2.23). Accordingly, the insertion speed was 0.1 mm/s slower with the picture-in-picture method.

### Hearing preservation

#### Patients

**Fig. 2:**
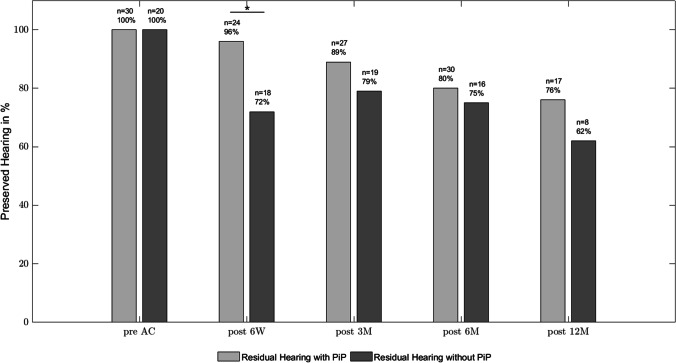
Residual hearing preservation over a 1-year follow-up-period. In the picture-in-picture group, the percentage of patients with residual hearing ranged from 96% 6 weeks postoperatively to 76% at the end of the follow-up period. In the without picture-in-picture group, 62% of patients showed residual hearing at the end of the follow-up period. Differences between groups were significant 6 weeks postoperatively (Chi-Square test, *p = *0.017, chi = 8.08). At 3 months (*p = *0.64, chi = 0.21), 6 months (*p = *0.69, chi = 0.15) and 12 months (*p = *0.46, chi = 0.52) postoperatively, no significant differences between groups were detected

Residual hearing was measured at 6 weeks, 3 months, 6 months and 12 months postoperatively. After 6 weeks postoperatively, hearing preservation was significantly better in the picture-in-picture group (96% versus 72% in the without picture-in-picture group, *p = *0.017). Hearing preservation during the follow-up period is shown in Fig. 2. Residual hearing preservation is not significantly better in patients implanted with intraoperative ECochG compared to the control group of patients implanted regularly without ECochG. However, after 3 months postoperatively there is a visible trend that the hearing thresholds are worse for patients without ECochG at 1000 Hz (Fig. 4).

#### Amplitude growth function of ECochG potentials

**Fig. 3:**
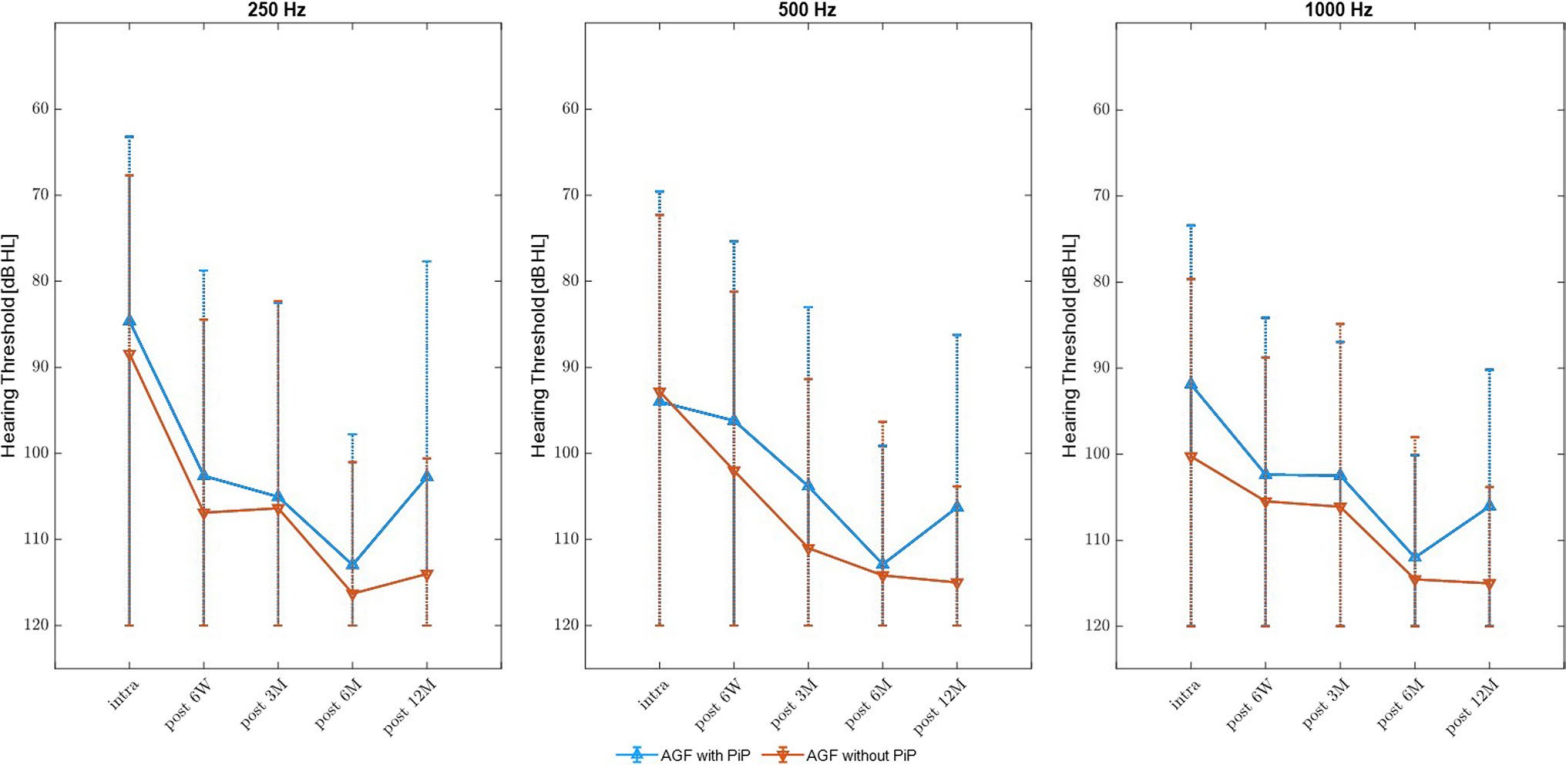
Results of AGF over a 1-year follow-up period. In the tested frequencies (250, 500 and 1000 Hz), AGF-results of the PiP-group showed a slightly better hearing threshold than the without PiP-group without reaching statistical significance (intraoperative: 250 Hz: *p = *0.46, *z* = 0.74; 500 Hz: *p = *0.83, *z* =  − 0,21; 1000 Hz: *p = *0.27, *z* = 1.09, 6 weeks: 250 Hz: *p = *0.55, *z* = 0.59; 500 Hz: *p = *0.35, *z* = 0.93; 1000 Hz: *p = *0.43, *z* = 0.78, 3 months: 250 Hz: *p = *0.83, *z* = 0.21; 500 Hz: *p = *0.18, *z* = 0.32; 1000 Hz: *p = *0.31, *z* = 1.01, 6 months: 250 Hz: *p = *0.29, *z* = 1.05; 500 Hz: *p = *0.45, *z* = 0.75; 1000 Hz: *p = *0.16, *z* = 1.39, 12 months: 250 Hz: *p = *0.35, *z* = 0.93; 500 Hz: *p = *0.39, *z* = 0.84; 1000 Hz: *p = *0.24, *z* = 1.16)

No significant differences between groups were detected with slightly better AGF thresholds in the PiP group. AGF-potentials at different frequencies over the follow-up-period are shown in Fig. 3.

#### Comparison of pure tone threshold and amplitude growth function

**Fig. 4:**
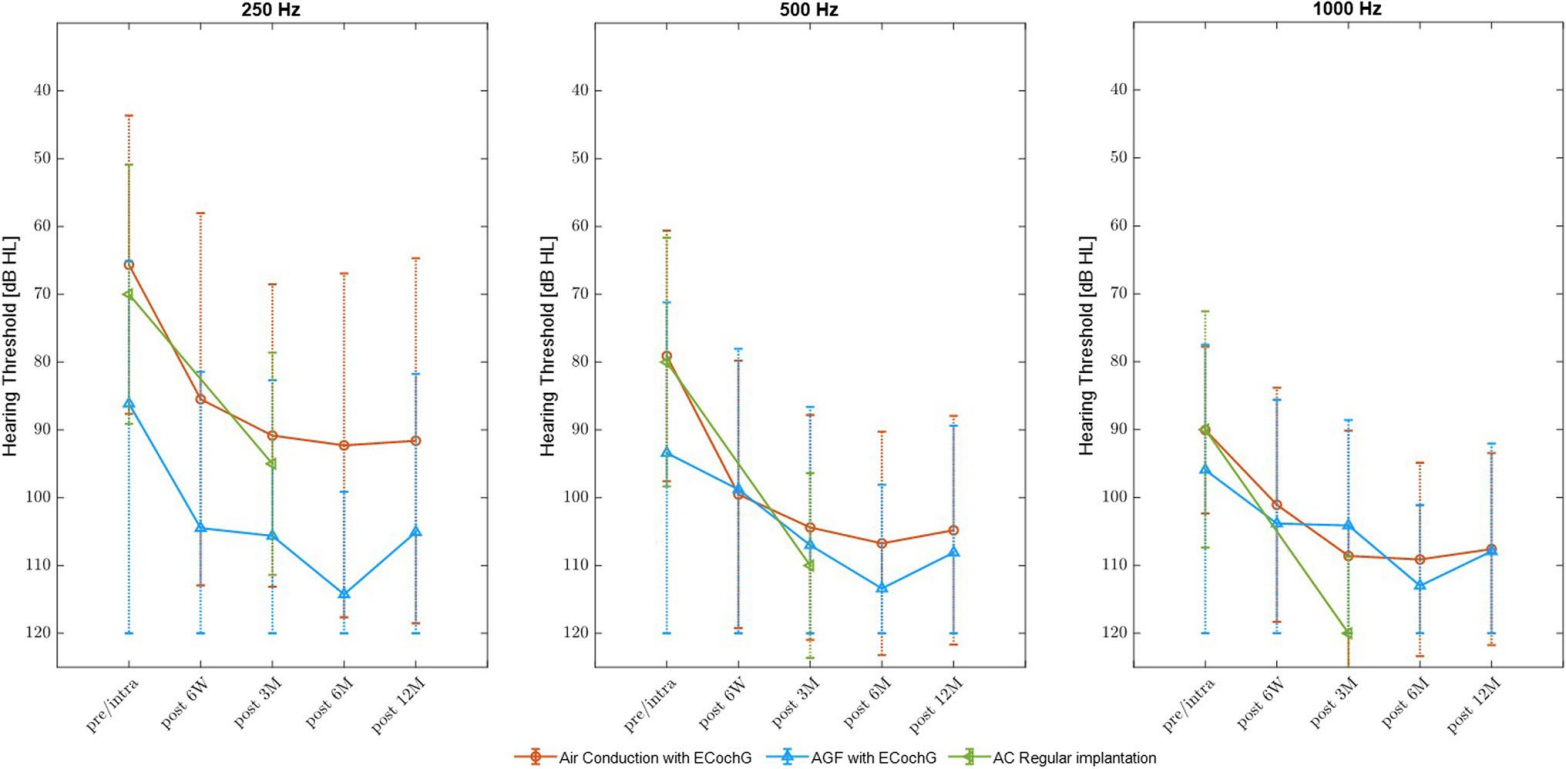
Pure tone thresholds and AGF over a 1-year follow-up period in patients with intraoperative ECochG- and without (regular implantation). At 250 Hz, AGF results differ significantly from pure tone thresholds over the follow-up period (6 weeks: *p* < 0.01, *z* = 3.61; 3 months: *p* < 0.01, *z* = 3.72; 6 months: *p* < 0.01, *z* = 5.4; 12 months: *p = *0.04, *z* = 2.04). At 500 Hz (6 weeks: *p = *1, *z* = 0; 3 months: *p* < 0.1, *z* = 1.61; 6 months: *p = *0.01, *z* = 2.5; 12 months: *p = *0.32, *z* = 0.97) and 1000 Hz (6 weeks: *p = *0.5, *z* = 0.6; 3 months: *p* < 0.24, *z* =  − 1.1; 6 months: *p = *0.05, *z* = 1.9; 12 months: *p = *0.76, *z* = 0.29), a significant difference was detected only after 6 months. Residual hearing preservation is non-significantly better in patients implanted with intraoperative ECochG compared to the control group of patients implanted regularly without ECochG, especially at 1000 Hz (pre/intraoperative: 250 Hz: *p = *0.53, *z* =  − 0.62; 500 Hz: *p* < 0.77, *z* =  − 0.29; 1000 Hz: *p = *0.4, *z* = 0.83; 3 months: 250 Hz: *p = *0.23, *z* =  − 1.19; 500 Hz: *p = *0.69, *z* = 0.39; 1000 Hz: *p = *0.59, *z* = 0.53)

For the evaluation and validation of AGF thresholds, a comparative analysis between the detected pure tone thresholds and the AGF results was calculated (Fig. 4). In addition, mean pure tone thresholds of 26 patients operated without intraoperative ECochG were used as a control group. It was shown that at 250 Hz, AGF results significantly differ from the pure tone thresholds of subjective audiometry at all times of evaluation (p < 0.01, *p = *0.04, respectively). For 500 and 1000 Hz, AGF results only differed significantly from the pure tone thresholds after 6 months postoperatively (*p = *0.01, *p = *0.05 respectively). Without the use of intraoperative ECochG, residual hearing after 3 months is worse than with the use of intraoperative ECochG measurements, a statistical significance could not be detected (Fig. 4).

#### Speech understanding

**Fig. 5:**
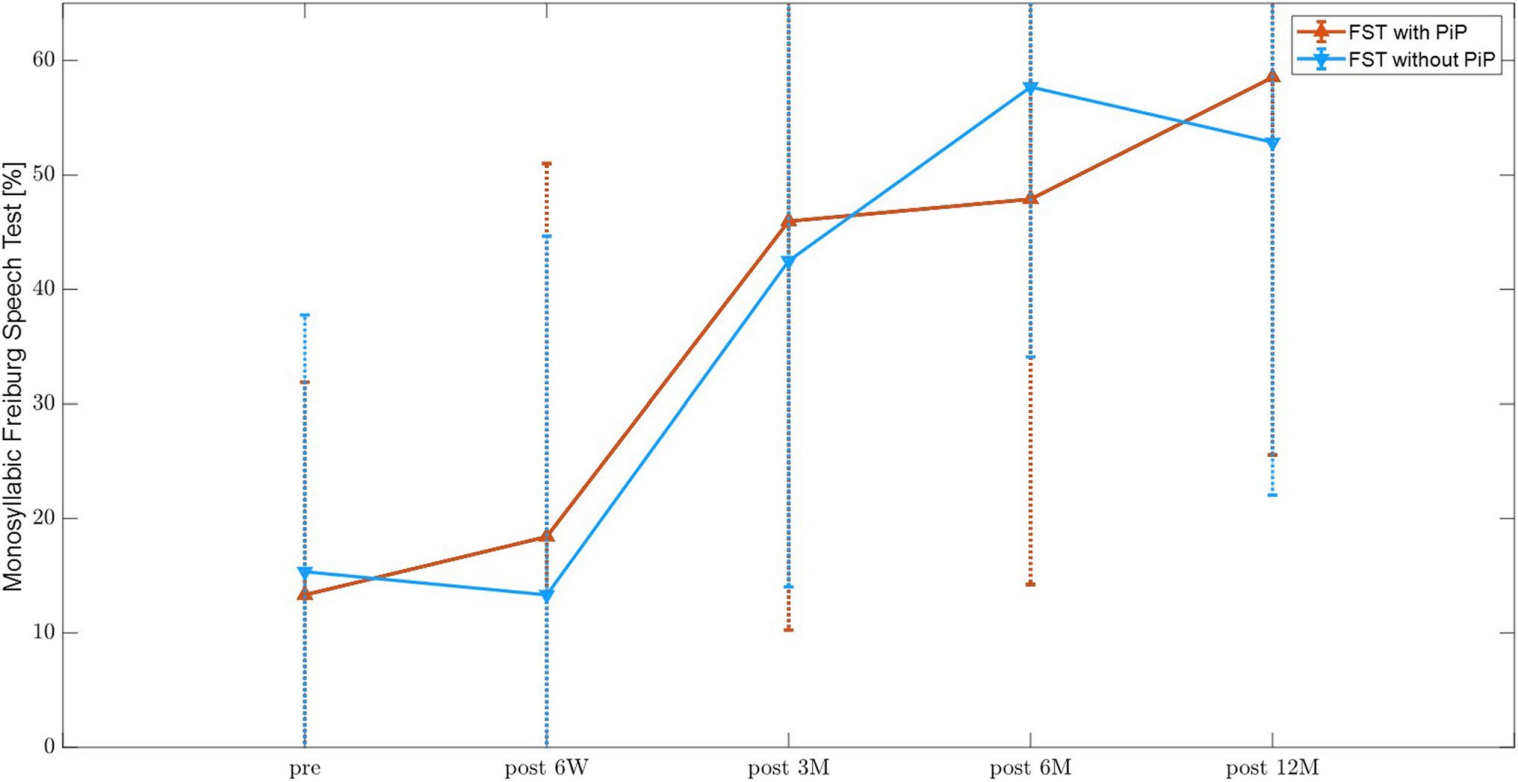
Speech understanding tested with Freiburger monosyllables before (best-aided condition) and after implantation. A significant improvement in speech understanding after one year compared to preoperative results was detected in both groups (PiP: *p* < 0.01, *z* =  − 3.72; wPiP: *p = *0.017, *z* =  − 2.39) without a significant difference between groups

For the evaluation of speech understanding Freiburger monosyllables were used (Fig. 5). Speech understanding at 65 dB improved by 38% in the without-picture-in-picture group and by 46% in the picture-in-picture group after one year compared to the best-aided condition preoperatively (p < 0.01, *p = *0.017 respectively). There was so significant difference between both groups (*p = *0.65, z = -0.48).

## Discussion

This is the first study analyzing digital real-time visualization of intraoperative ECochG potentials during cochlear implantation in a large patient cohort. The goal of this randomized prospective trial was to evaluate patients´ hearing outcomes focusing on the preservation of residual hearing. We were specifically aiming at comparing postoperative audiometric results between two groups. In group I intraoperative ECochG signals were visualized in the surgeon´s field of view (picture-in-picture). In group II intraoperative ECochG signals were only acoustically reported by the audiologist (without picture-in-picture). In this study, residual hearing preservation in low-frequency range was achieved in 76% of the patients. Thereby, direct visualization of EcochG led to an improved residual hearing preservation. Taken together we think that digital real-time visualization of intraoperative EcochG during cochlea-implantation is a promising tool to improve patients´ hearing outcome.

Different methods to test for the correct position of the electrode immediately after or during surgery have been established. Electrically evoked compound action potentials such as e.g. NRT^®^ (neural response telemetry) are commonly performed but do not provide real-time information during insertion. They are mostly used as a post-insertion control instrument and for objective fitting. Moreover, stimulation is not performed via tone burst but by electric activation of the implant [13] and therefore is not helpful to preserve residual acoustical hearing. In contrast, ECochG can provide real-time feedback to the surgeon during electrode insertion. However, until now, there are two major problems with ECochG performed during cochlear implant surgery.

Firstly, the majority of the present studies assessing intraoperative ECochG measurement are not based on real-time feedback during insertion but rather provide post-insertional feedback although potentials are measured immediately after insertion [20, 24]. With this approach, relevant information during the insertion period may be lost. In a model experiment performed by a senior cochlear implant surgeon, the mean insertion speed was 0.48 mm/sec with a mean insertion time between 44.4 and 48.6 s [25]. Another retrospective analysis of 54 uneventful insertions revealed a mean insertion speed of 96.5 mm/min [26]. Although the insertion period seems relatively short, manual insertion by the surgeon at a constant speed without trembling is limited. In our opinion, the insertion period needs to be considered as the key time window during which changes in insertion speed or -angle can be taken if drops in ECochG potentials occur aiming at avoiding intracochlear trauma with the need for immediate feedback. Secondly, even if direct feedback is given, this needs to be done – up to now—verbally by the audiologist or acoustically by the machine performing the measurement. Considering external factors such as human reaction rate, high noise levels and the presence of several team members in the operating room, the surgeon operating via microscope has multiple tasks to fulfill simultaneously in the moment of electrode insertion. Thus, relevant information come along with a considerable time delay which in turn prohibits adequate reaction, e.g. short pause of electrode insertion or gentle pull back.

In this analysis, it was demonstrated that digital real-time visualization of intraoperative ECochG with a digital microscope facilitates this feedback and can add to hearing preservation. An important finding was, that in the picture-in-picture group the insertion speed was lower than in the without picture-in-picture group. This can be explained by continuous monitoring of the ECochG-curve during electrode insertion leading to a slower insertion. Residual hearing preservation in low-frequency range was possible in 76% of the patients (picture-in-picture group). These data are roughly in line with a recent investigation assessing residual hearing after cochlear implantation with a new lateral wall electrode in a cohort of 20 patients where intraoperative ECochG was used [27]. Another study of 17 patients focusing on residual hearing preservation obtained comparable results [28]. However, there are data that indicate a lower rate of residual hearing preservation than in our investigation [29]. Of note, in that study, intraoperative ECochG was not performed. We think that the use of intraoperative ECochG and especially its direct visualization will emerge as an essential part of modern cochlear implant surgery. This is underlined by our comparative analysis, which yielded better hearing preservation in patients operated with ECochG compared to no use of ECochG at all.

Regarding the results of the AGF of ECochG-potentials in the follow-up period, it was shown that AGF potentials were lower in the picture-in-picture group over all frequencies. This also speaks for real-time visualization of ECochG-potentials during insertion. However, so far, there are still lots of unanswered questions concerning AGF-potentials during cochlear implantation especially due to the heterogeneity of ECochG monitoring technique [30]. Comparing AGF thresholds with pure tone audiometry, AGF potentials at 250 Hz were significantly lower over the whole follow-up period than thresholds obtained by pure tone audiometry. Studies investigating on AGF potentials showed that they indicate neural and hair cell function and that postoperative ECochG thresholds positively correlate with behavioral thresholds after surgery [31]. However, in the respected study, this could only be confirmed for 500 and 1000 Hz but not for 250 Hz. Although a specific explanation for the phenomenon cannot be given, these new data could add to a better understanding of intraoperative ECochG potentials. A better speech understanding in the picture-in-picture group further underlined the positive effect of direct visualization of intraoperative ECochG enabling the surgeon to directly change insertion speed or angle if drops in the curve occur.

Taken together, this study demonstrates valid data that indicate a positive effect on residual hearing preservation in cochlear-implant surgery when digital live imaging of intraoperative ECochG is performed. In the future, ongoing close cooperation of scientists, surgeons and technicians will contribute to technical progress and digitalization in otologic surgery. Robot-supported cochlear implant surgery providing a slow insertion process at constant speed is an emerging field that might benefit from digital live visualization of intraoperative ECochG in the future [32].

## Data Availability

Not applicable.
